# Effects of Marine Oils, Digested with Human Fluids, on Cellular Viability and Stress Protein Expression in Human Intestinal Caco-2 Cells

**DOI:** 10.3390/nu9111213

**Published:** 2017-11-04

**Authors:** Cecilia Tullberg, Gerd Vegarud, Ingrid Undeland, Nathalie Scheers

**Affiliations:** 1 Division of Food and Nutrition Science, Department of Biology and Biological Engineering, Chalmers University of Technology, Kemigården 4, 412 96 Gothenburg, Sweden; undeland@chalmers.se; 2 Division of Food Proteins, Structure and Biological Function, Department of Chemistry, Biotechnology and Food Science, Norwegian University of Life Sciences, Chr. M. Falsens vei 1, 1432 Ås, Norway; gerd.vegarud@nmbu.no

**Keywords:** Caco-2, human digests, lipid oxidation, marine oil, HHE, MDA, Trx-1, HSP-70

## Abstract

In vitro digestion of marine oils has been reported to promote lipid oxidation, including the formation of reactive aldehydes (e.g., malondialdehyde (MDA) and 4-hydroxy-2-hexenal (HHE)). We aimed to investigate if human in vitro digestion of supplemental levels of oils from algae, cod liver, and krill, in addition to pure MDA and HHE, affect intestinal Caco-2 cell survival and oxidative stress. Cell viability was not significantly affected by the digests of marine oils or by pure MDA and HHE (0–90 μM). Cellular levels of HSP-70, a chaperone involved in the prevention of stress-induced protein unfolding was significantly decreased (14%, 28%, and 14% of control for algae, cod and krill oil, respectively; *p* ≤ 0.05). The oxidoreductase thioredoxin-1 (Trx-1) involved in reducing oxidative stress was also lower after incubation with the digested oils (26%, 53%, and 22% of control for algae, cod, and krill oil, respectively; *p* ≤ 0.001). The aldehydes MDA and HHE did not affect HSP-70 or Trx-1 at low levels (8.3 and 1.4 μM, respectively), whilst a mixture of MDA and HHE lowered Trx-1 at high levels (45 μM), indicating less exposure to oxidative stress. We conclude that human digests of the investigated marine oils and their content of MDA and HHE did not cause a stress response in human intestinal Caco-2 cells.

## 1. Introduction

Intake of marine omega-3 fatty acids, i.e., the long-chain *n*-3 polyunsaturated fatty acids (LC *n*-3 PUFA) eicosapentaenoic acid (EPA), and docosahexaenoic acid (DHA), has been associated with beneficial health effects related to e.g., cardiovascular diseases [1,2]. Intake of marine oils high in EPA and DHA as dietary supplements, rather than ingesting them as a part of a complex seafood diet, has raised concerns regarding the stability of the pure oils, i.e., oils being separated from their native matrix. Marine oils are highly susceptible to peroxidation during storage and processing, and there are also indications of that the oils oxidize during gastrointestinal (GI) digestion [3,4,5], which is supported by several review [4,5], in vitro [3,6,7,8,9], and animal studies [10] of simulated GI digestion of marine oils.

Malondialdehyde (MDA) is one of the most well-known lipid oxidation products that is formed from PUFA. MDA is often used as a biomarker for lipid oxidation [11,12,13,14]. MDA has been attributed possible genotoxic features due to its ability to crosslink proteins and DNA, and it has also been associated with the development of cardiovascular disease, as reviewed by Del Rio et al. [15], and Uchida [16]. α,β-unsaturated aldehydes are thought to be more toxic than MDA, and are more reactive toward nucleophiles due to the hydroxyl-group, which is positioned close to the double bond [13,17]. One such aldehyde is 4-hydroxy-2-hexenal (HHE), which is derived from *n*-3 PUFAs [18,19], and is hence an important endpoint in the investigation of oxidative stability of marine oils.

Fish oil has been associated with anti-inflammatory properties, such as the down-regulation of inflammatory cytokines (e.g., TNF-α, IL-6), the increase of cellular membrane content of EPA and DHA, and the decrease of cellular membrane content of arachidonic acid (AA), all in healthy humans [20]. Also, EPA and DHA supplementation has led to decreased T-cell reactivity in cell and animal studies [20]. In murine models, high fat LC *n*-3 PUFA diets have been observed to decrease the levels of inflammation markers in plasma (IL-6 and MCP-1) [21] and in the spleen (NF-κB) [22], the same markers that increased in the study where mice were fed with an oxidized diet [10]. In another murine study, in which the mice were fed an oxidized LC *n*-3 PUFA diet, inflammatory markers such as NF-κB and glutathione peroxidase increased [10]. In addition, it was observed that HHE given orally to the mice was associated with an inflammatory response, as well as the formation of HHE-adducts. HHE in plasma increased significantly in mice given the oxidized LC *n*-3 PUFA diet [10]. In the search for sustainable LC *n*-3 PUFA rich substitutes to the traditional cod liver oil and fish oil [23,24], krill and microalgae oil (in this article referred to as algae oil) are two plausible candidates. Krill oil contains more EPA when compared to algae oil, while algae produce its own DHA, and therefore the oil is richer in this fatty acid (FA) [25]. They are both high in naturally occurring antioxidants, e.g., astaxanthin in krill oil [25,26], and phenolic compounds, flavonoids, sterols, and β-carotene in algae oil [27]. A specific feature of krill oil is that most of the LC *n*-3 PUFA are bound in phospholipids. In microalgae, fish muscle, and liver, the LC *n*-3 PUFA are incorporated in triacylglycerols (TG).

In this study, we investigated the effects of in vitro digests of three marine oils (cod liver oil, krill oil, and algae oil) generated with human digestive fluids on a cultured human intestinal epithelium (Caco-2 cell line). We measured the content of the secondary oxidation products MDA and HHE in the digests, and exposed the epithelium to pure MDA and HHE at these levels and above. We also measured the aldehyde levels in the basal medium. In addition, we examined stress-related protein levels in the Caco-2 cells with proteome profiler arrays to evaluate if the cells were exposed to stress during the various treatments.

## 2. Materials and Methods

### 2.1. Materials

The pre-cursor for MDA standard 1,1,3,3-tetraethoxypropane (TEP), 2,4-dinitrophenylhydrazine (DNPH), and reagents used for cell experiments were all purchased from Sigma-Aldrich (Schnelldorf, Germany). 4-hydroxy-2-hexenal (HHE), 4-hydroxy-2-nonenal (HNE), and 4-oxy-2-nonenal (ONE) standards were supplied from Cayman Chemicals (Ann Arbor, MI, USA). Media and supplements that were needed for cell culture maintenance were purchased from PAA (Pasching, Austria), and disposables in polystyrene used for cell cultivation and maintenance were bought from Corning (San Francisco, MA, USA). The human cell stress array kit was purchased from Bio-techne (Abingdon, UK).

### 2.2. Collection of Human Digestive Fluids

Saliva was collected from seven healthy fasting volunteers at Chalmers University of Technology (Gothenburg, Sweden) in November 2015. Saliva was collected in the morning by sterile straw pipets (Kemikalia; Skurup, Sweden) and volunteers were shown pictures of fish dishes to stimulate spontaneous drooling during collection. Saliva was pooled to eliminate individual effects, centrifuged, and stored at −80 °C.

Human gastric juice (HGJ) and human duodenal juice (HDJ) were aspirated from six healthy volunteers at Lovisenberg Diakonale Hospital (Oslo, Norway), as described by Ulleberg et al. [28], and Holm et al. [29]. The volunteers were semi-fasting using a stimulatory solution and aspiration was done using gastroscopy and a triple lumen tube (Maxter Catheters, Marseille, France), aspiration details are further described by Ulleberg et al. [28]. Aspirates were pooled, enzyme activities, and pH of the human GI fluids were recorded according to Minekus et al. [30], and the HGJ and HDJ were stored separately at −80 °C. All of the participants in the study were volunteers with informed consent, and the study was performed according to the Declaration of Helsinki. Ethical approval was received from the Norwegian Regional Ethics Committee (project no. 2012/2230, Biobank no. 2012/2210).

The human digestive fluids were characterized according to Minekus et al. [30], and enzymatic activities were measured in connection to the in vitro digestions. The pepsin activity of HGJ was 1200 U/mL, the gastric lipase activity in the HGJ was 16 U/mL, the pancreatic lipase activity in the HDJ was 48 U/mL, and the bile salt concentration in the HDJ was 0.230 mM. Ascorbic acid was analyzed by the method described by Lykkesfeldt et al. by ion chromatography followed by electrochemical detection [31], and approximately 0.3 ppm was detected in both HGJ and HDJ. Ca^2+^ was analyzed by an ion chromatograph couples with UV-vis according to Fredrikson et al. [32], and was found to be present in HGJ at 35 ppm and in HDJ at 16 ppm.

### 2.3. Marine Oils

Refined cod liver oil (*Gadus morhua*), without added antioxidants, was supplied by Lýsi hf (Reykjavík, Iceland). Unrefined algae oil from *Schizochytrium* sp. called Life’s DHA S35-CO100 was supplied from DSM (Basel, Switzerland). Unrefined krill oil from Antarctic krill (*Euphausia superba*) called Superba^™^ Krill Oil (Aker Biomarine Antarctic AS, Oslo, Norway) was provided by Sanpharm AB (Gothenburg, Sweden). The LC *n*-3 PUFA profile of the oils, % as reported by Jónsdóttir et al. and according to the manufacturers specification [33], and quantitatively (mg FAME/g oil) measured in-house according to Cavonius et al. [34], can be found in the supplementary material (Table S1).

### 2.4. In Vitro Digestion with Human Digests

The three marine oils were digested in vitro in a static three-step digestion model, using the human digestive fluids. The model is based on the standardized InfoGest protocol with minor modifications [30]. The recommended daily intake (RDI) for supplemental oils are based on the consumption of EPA and DHA, and therefore the dose of each oil was normalized to its EPA and DHA content before the simulated GI digestion. For each oil, an amount providing 5 mg total LC *n*-3 PUFA, i.e., EPA+DHA, was used, which corresponds to 250 mg on a human level. Water was added to achieve samples of the same volume. In control digestions, oils were omitted and only water was used. In short, digestion was performed in darkness and the oil-water mixture was digested by one volume saliva, followed by HGJ addition (1:1, pH 6, 37 °C, 50 rpm, 120 min), including adjustment of pH to pH 3 after 60 min. Intestinal digestion was performed by the addition of HDJ (1:1, pH 7, 37 °C, 250 rpm, 90 min). Digested samples were flushed with N_2_ gas (15 s) and stored in −80 °C until aldehyde analysis and cell experiments.

### 2.5. Cell Line

Caco-2 cells (HTB-37), passage 19, were obtained from the American Type Culture Collection (Rockville, MD, USA). The cells were cultured in an incubator at 37 °C/5% CO_2_/95% humidified air. The medium used was EMEM (FBS; 10%) supplemented with Normocin^™^ (0.2%; Invivogen, San Diego, CA, USA). The medium was replaced every second or third day and passaging of cells was done at approximately 80% confluence. At passage 29–37, the cells were seeded in 12-well plates with Transwell^®^ polycarbonate inserts (0.4 μm; Corning, San Francisco, MA, USA) at 60,000 cells/insert or without inserts (CellBiND^®^, polystyrene; Corning, Kennebunk, ME, USA) at 200,000 cells/well. All of the experiments were carried out 14 days post-seeding.

### 2.6. Cell Experiments

Cells (on inserts) were treated with in vitro digested marine oils and control digests (without oil), diluted 1:1 in the apical medium. The apical medium was added (0.5 mL) to the cells 24 h prior to the experiments to let the cells produce endogenous trypsin inhibitor. At the time of the experiment, 0.25 mL of the apical medium was replaced by 0.25 mL of the digests and were then left in the incubator for 2 h (37 °C). Controls with only medium were included, as well as standards with MDA in water and HHE in DMSO (DMSO at 0.01%), corresponding to the highest levels detected in digests; 16.6 and 2.8 μM, respectively. Standards were as digests diluted 1:1 in the apical medium, hence exposure of cells to MDA and HHE were 8.3 and 1.4 μM, respectively. From here on, these levels of MDA and HHE are referred to as “low” levels. To separately study the toxicity of the aldehydes at different levels, an experiment with cells in wells without inserts was performed. MDA levels tested were 8.3, 45, and 90 μM; HHE levels tested were 1.4, 45, and 90 μM. A mix (1:1) of the two aldehydes was also studied at (1) 45 μM each of MDA and HHE; (2) 22.5 μM each of MDA and HHE, and (3) 4.15 μM MDA and 0.7 μM HHE, to test combined, e.g., synergistic, effects. 90 μM of the individual aldehydes and 45 μM of each in a mix are from here on referred to as “high” levels. The highest aldehyde levels are in the same range, as previously used by Awada et al. [10], the levels in the middle are half of the highest levels, and in the same range as used by Alghazeer et al. [35]. Minimum Essential Medium Eagle, HEPES modification powder (14.2 g/L; Sigma-Aldrich, Schnelldorf, Germany) was used to be able to achieve high MDA concentrations without dilution, and mixed (1:1) with the ordinary medium that was used when studying the mixed effect of MDA and HHE. After the 2 h of incubation, the medium was aspirated and the cells were washed in PBS and lysed.

### 2.7. Harvesting of Caco-2 Cells and Protein Analysis

The medium was removed and the cells were washed in PBS prior to harvest. The basal medium was collected and the cells were lysed in RIPA (Sigma-Aldrich, Schnelldorf, Germany) with EDTA-free Pierce™ Protease and Phosphatase inhibitor (Thermo Fisher Scientific, Waltham, MA, USA). Total cellular protein content was measured by Pierce™ BCA Protein Assay Kit (Thermo Fisher Scientific, Waltham, MA, USA), following the instructions from the manufacturer. From the total protein content (which is proportional to cell number), cell viability/survival was estimated.

### 2.8. Analysis of Peroxide Value (PV) and Aldehydes (HHE & MDA)

Peroxide value (PV) was analysed prior to digestion in the crude oils by thiocyanate and ferric iron complexation, according to Undeland et al. [36].

HHE and MDA were analyzed, as described by Tullberg et al. [8]. Briefly, digests and basolateral media were acidified to precipitate proteins, followed by DNPH-derivatization and dichloromethane extraction. Samples were evaporated and re-suspended in MeOH, aldehydes were then determined in digests by detection on LC/APCI-MS (Agilent 1260 HPLC coupled with Agilent 6120 quadrupole; Agilent Technologies, Waldbron, Germany) in negative mode, using external standards for MDA and HHE. Analysis of the data was carried out using the software Agilent ChemStation (Agilent Technologies, Böblingen, Germany).

### 2.9. Human Cell Stress Array Analysis

The Human Cell Stress Array Kit (Bio-techne, Minneapolis, MN, USA) was used according to manufacturer’s protocol. Briefly, membranes coated with 26 capture antibodies were blocked (1 h, RT). The blocking buffer was aspirated and the samples were adjusted by total cellular protein content were added (105 μg total protein; *n* = 3) together with a biotinylated antibody cocktail for detection (overnight, 130 rpm, 4 °C). Membranes were then washed in a wash buffer (10 min, repeated three times). Streptavidin-conjugated horseradish peroxidase (HRP) was added to the membranes (30 min, RT), and membranes were again washed (10 min, repeated 3 times). After the last washing step, a reaction mixture containing hydrogen peroxide and luminol (1:1) was added to the membranes, and was instantly analyzed with a detection system for chemiluminescence (Chemidoc XRS+, Bio-Rad), followed by software analysis of the images by ImageLab (Bio-Rad). See Table 1 for the specific analytes detected.

### 2.10. Statistics

Calculated values are presented as mean values ± standard deviation (SD; *n* = 3) or when *n* = 2 as mean values ± (max − min)/2. Digestion of oils with subsequent cell experiments were made in triplicates and repeated at three occasions, human stress arrays were done in duplicates and repeated 2–3 times. The significance of the difference between treatment and control was analyzed by Student’s two-tailed, unpaired *t* test, and treatments were compared by a one- or two-way analysis of variance (ANOVA; Microsoft Office Excel, 2013), followed by treatment to treatment *t* test as above, whenever applicable. Differences were considered significant at *p* ≤ 0.05. Significant levels are denoted in the graphs and tables when applicable; * = *p* ≤ 0.05, ** = *p* ≤ 0.01, *** = *p* ≤ 0.001.

## 3. Results

### 3.1. MDA and HHE Formation during In Vitro Digestion with Human Digestive Fluids

The initial aldehyde levels prior to digesteion were 0.013 ± 0.01, 0.11 ± 0.05, and 0.38 ± 0.14 μM, for MDA, and 0.005 ± 0.009, 0.17 ± 0.02, and 0.04 ± 0.007 μM for HHE in the algae-, cod liver- and krill oil, respectively. The corresponding peroxide values (PV) in the crude oils were 0.18 ± 0.05 in algae-, 1.48 ± 0.06 in cod liver- and 1.00 ± 0.30 (mmol/kg oil) in the krill oil. According to the PV and HHE measurement, the cod liver oil was the most oxidized oil initially, however the krill oil contained a higher initial concentration of MDA. The aldehydes MDA and HHE both increased from start (*t* = 0 min) to end (*t* = 210 min) of the in vitro digestion. The levels of MDA and HHE detected in the digests were approximately 4 and 7–20 times higher in the cod liver oil as compared to the other oils, respectively (Table 2).

### 3.2. Cell Survival Was Not Significantly Affected by Either Oil Digests or HHE and MDA

**Figure 1:**
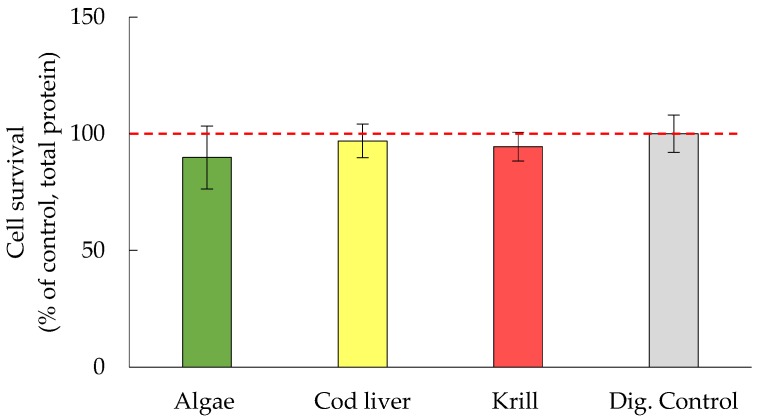
Cell survival estimated from the change in total protein level between untreated and treated cells, data are shown as mean % ± (max − min)/2, *n* = 2. Dig. Control = digests from digestion of only water.

**Figure 2:**
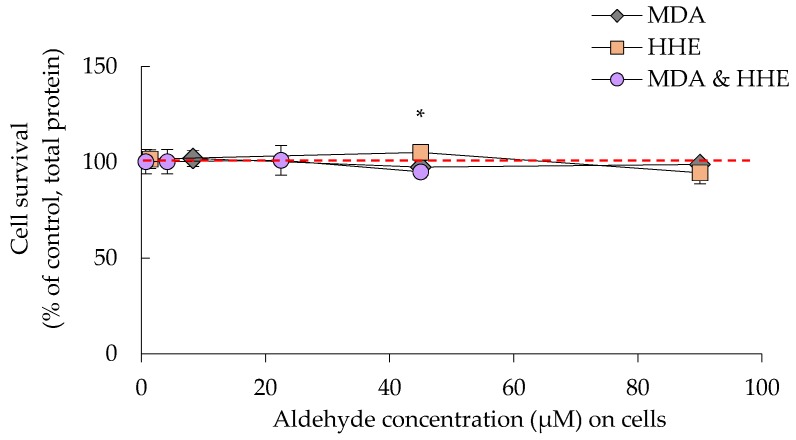
Cell survival in the presence of increasing concentrations of the aldehydes malondialdehyde (MDA) and 4-hydroxy-2-hexenal (HHE), data are shown as means ± (max − min)/2, *n* = 2, or *n* = 4 for the lowest concentrations of MDA (8.3 μM) and HHE (1.4 μM). Significant difference (*p* ≤ 0.05). MDA and HHE is the combination of both aldehydes, at the lowest concentrations, 4.15 μM MDA and 0.7 μM HHE. * = *p* ≤ 0.05.

There was no significant effect of digested oils on cell survival after 2 h of incubation and 22 additional hours with fresh medium (Figure 1). In a dose-response experiment with the oxidation products MDA and HHE, similar results were achieved with no adverse effect on cell viability (Figure 2). A minor increase was seen in protein levels when adding 45 μM of HHE, however this effect was within 2 SD:s and thus natural variation.

### 3.3. Cellular Levels of HSP-70 and Trx-1 Were Decreased in the Presence of Digested Marine Oils

**Figure 3:**
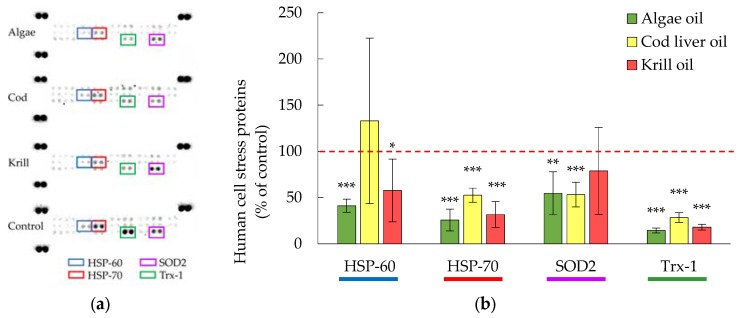
(**a**) Representative membranes showing the expression of human stress-related proteins (Proteome profiler™ arrays) in the presence of marine oils digested by human GI fluids.; (**b**) Bar graph representation of data for pooled triplicates of lysates ± SD, *n* = 3. * = *p* ≤ 0.05, ** = *p* ≤ 0.01, *** = *p* ≤ 0.005. HSP-60= Heat Shock Protein-60; HSP-70= Heat Shock Protein-70; SOD2= Superoxide dismutase 2; Trx-1= Thioredoxin-1.

All of the digested oils significantly decreased the expression of HSP-70 and Trx-1 (*p* ≤ 0.001), Figure 3. In addition, SOD2 levels were significantly lowered in the presence of algae and cod liver oil digests. Krill oil digests did not significantly affect SOD2 levels. HSP-70, Trx-1 and SOD2 are all a part of the anti-oxidative stress defense and are generally increased when the cells are exposed to oxidative stress [37,38]. HSP-70 is a chaperone protein that reduces oxidative damage by binding to proteins, which prevents unfolding and aggregation. Trx-1 is an oxidoreductase facilitating the reduction of oxidized proteins and SOD2 is a superoxide dismutase that eliminates superoxide radicals. The levels of HSP-60, coming from the same family as HSP-70, was significantly lowered in the presence of digested algae and krill oil, however not by digested cod liver oil.

### 3.4. Cellular Hsp-70 and Trx-1 Levels Were Not Affected by Low MDA and HHE Levels (8.3 and 1.4 μM)

**Figure 4:**
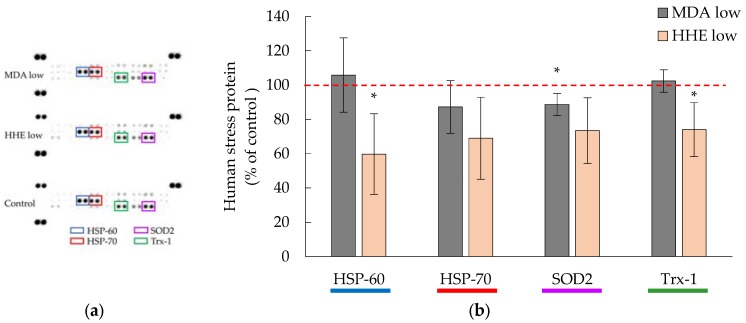
(**a**) Representative membrane showing the expression of human stress-related proteins (Proteome profiler™ arrays) in the presence of “low” aldehyde levels, 1.4 μM 4-hydroxy-2-hexenal (HHE) and 8.3 μM malondialdehyde (MDA), i.e., the levels found in cod liver oil digests; (**b**) Bar graph of the data for pooled triplicates of lysates ± (max − min)/2, *n* = 2. * = *p* ≤ 0.05. HSP-60= Heat Shock Protein-60; HSP-70= Heat Shock Protein-70; SOD2= Superoxide dismutase 2; Trx-1= Thioredoxin-1.

Low concentrations of MDA (8.3 μM) and HHE (1.4 μM), corresponding to the highest aldehyde levels in the marine oil digests, which the cells were exposed to, did not, or did only slightly, affect the cellular levels of HSP-70, TRX-1, SOD2, Figure 4. In addition, the HSP-70 family member, HSP-60, decreased significantly (*p* = 0.043) in the presence of low level HHE (1.4 μM).

### 3.5. High Levels (90 μM) of MDA and HHE Did Not Affect the Cellular Levels of HSP-70 and Trx-1

**Figure 5:**
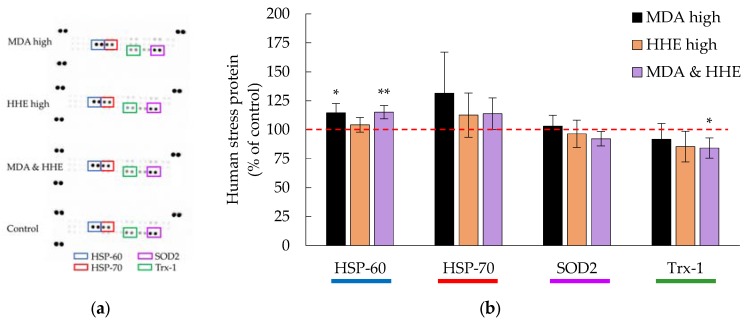
(**a**) A representative membrane showing the expression of human stress-related proteins (Proteome profiler™ arrays) in the presence of high aldehyde levels (90 μM MDA; 90 μM HHE; 45 μM MDA & HHE); (**b**) Bar graph of the data for pooled triplicates of lysates of cells in the presence of MDA (90 μM), HHE (90 μM) and a mix of MDA and HHE (45 μM each), data are shown as means ± (max − min)/2, *n* = 2. * = *p* ≤ 0.05, ** = *p* ≤ 0.01. MDA= malondialdehyde; HHE= 4-hydroxy-2-hexenal; HSP-60= Heat Shock Protein-60; HSP-70= Heat Shock Protein-70; SOD2= Superoxide dismutase 2; Trx-1= Thioredoxin-1.

High concentrations of aldehydes (90 μM), did not significantly affect the cellular levels of HSP-70 and Trx-1, Figure 5. The stress proteins detected were even closer to 100% of the control as compared to the addition of low aldehyde levels to the cells, but HSP-60 significantly increased in the presence of high MDA concentrations (*p* = 0.026). Testing a combination of MDA and HHE, both at 45 μM, the result was a combined effect that was similar to the presence of isolated aldehydes at 90 μM. HSP-60 however, increased (*p* = 0.0054, MDA & HHE) when compared to control, and Trx-1 was significantly lower than the control when cells were exposed to the MDA and HHE mix (*p* = 0.032).

### 3.6. Levels of MDA and HHE on the Basal Side of the Epithelium

**Figure 6:**
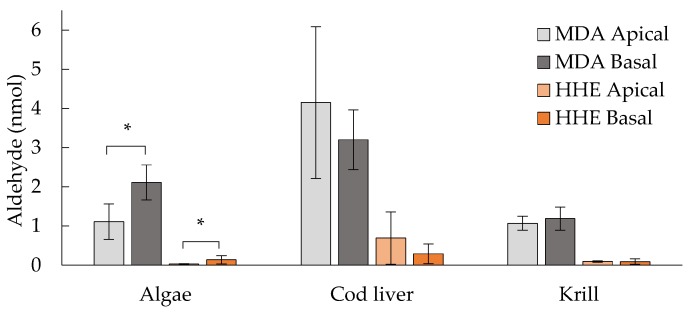
Aldehyde (nmol) added to the apical medium and detected in the basal medium of Caco-2 cells after incubation (*t* = 2 h). Data are shown as means ± standard deviation (SD), *n* = 3. * = *p* ≤ 0.05. MDA= malondialdehyde; HHE= 4-hydroxy-2-hexenal.

High concentrations of MDA and HHE were found in the basal medium after the incubation of Caco-2 cells with the oil digests (1.4, 2.1 and 0.8 μM MDA; 0.09, 0.2 and 0.06 μM HHE for algae, cod liver, and krill oil, respectively). The absolute amounts (nmol) in the apical versus the basal medium are presented in Figure 6. The medium with the algae oil digests contained significantly (*p* ≤ 0.05) more MDA and HHE in the basal medium when compared to the apical medium. With the present experimental setup, we do not know if, or how much of, MDA and HHE that was actually transported across the intestinal epithelium. The decomposition of the digested oils is expected to be extensive, due to the elevated temperature (T = 37 °C), during the 2 h incubation time. This is expected to give increasing levels of aldehydes in the apical medium, and therefore a ratio of the basal concentration of MDA and HHE to the added sample concentration of MDA and HHE will not give an accurate measure of the transport. Thus, the bioavailability cannot be correctly estimated. Awada et al., who used deuterium labelling, estimated the basolateral transport of HHE in Caco-2/TC7 cells, which resulted in approximately 0.2% HHE transfer (100 μM, 24 h) [10]. This gives an indication of in which magnitude we should expect to find the transport. When we conducted a transport experiment with pure HHE, we found that HHE was transported across the epithelium at 1.14% when incubated with 1.4 μM HHE for 2 h. Corresponding data was 10.6% when using the lower concentration of HHE (0.65 μM, 2 h), Table 3.

## 4. Discussion

### 4.1. Oxidation of the Oils during In Vitro Digestion

Both MDA and HHE levels increased during in vitro digestion with human digestive fluids. These results are in agreement with results from studies where cod liver oil and salmon were subjected to in vitro digestion models, in which enzymes and bile have been of porcine or other animal origin [9,39,40]. Increase in aldehyde formation during digestion was also observed in our previous study, when digesting cod liver oil with human GI fluids [8].

### 4.2. Human Digests of Marine Oils and the Aldehydes MDA and HHE Had No Adverse Effect on the Epithelium

From these studies, we can conclude that the digested oils and their MDA/HHE content did not have a negative impact on the cells. The absence of effects of pure MDA and HHE (1.4–90 μM) on cell survival is supported by Awada et al. [10], in which no effect in TEER after incubating Caco-2 and T27 cells with similar levels of aldehydes (24 h, 0–100 μM) was observed. On the contrary, proteins associated with oxidative damage (Trx-1 and HSP-70) were down-regulated in the presence of all three digested marine oils, suggesting that the cells actually were experiencing less oxidative stress than in the absence of the digested oils. In addition, pure MDA and HHE, corresponding to the concentrations found in the oil digests, did not increase oxidative stress markers in the cells, HHE even significantly reduced the levels of Trx-1 in the cells, indicating that MDA and HHE had no harmful effect at the levels found after in vitro digestion of the three marine oils with human GI fluids. Hence, the lipid oxidation taking place during in vitro GI digestion in this study was not associated with harmful effects in human intestinal cells. Although it is not directly comparable with studies of digests in intestinal cell models, HHE has been shown to promote Nrf2 activation in HUVEC cells [41], and also, oxidized EPA has been shown to inhibit NF-κB activation in murine aortic endothelial cells [42]. In addition, a recent review by Roy et al. suggests that ROS play an important role for cellular redox homeostasis [43]. All of these data are in line with our findings. However, there are also some studies with digested marine oils showing opposite results to ours regarding lipid oxidation and effects on Caco-2 cells [7,10]. Different outcomes may in several cases be a direct cause of different experimental setups, such as exposing intestinal cells to undigested oils, exposing other cell types than intestinal cells to GI digests, using oils with a higher oxidation degree, using longer incubation time, or feeding a cell type of non-intestinal origin with oils. Even though we did not identify the adverse effects of MDA and HHE on the intestinal level, there may be potentially harmful systemic effects of lipid oxidation products, e.g., MDA protein adducts have been implicated in coronary artery disease development [44].

### 4.3. Comparison of the Different Marine Oils

In the crude oils, the concentration of EPA plus DHA was in the following order: algae > krill > cod liver oil. Digests with cod liver oil therefore contained the highest amount (mg/mL) of total oil per digest when compared to the other oils (10% more oil than krill oil, 50% more than algae oil). Algae oil contained mainly DHA, while the other two oils contained ratios between EPA and DHA that were 0.8 and 2.0 for cod liver oil and krill oil, respectively. The krill oil is unique in that it contains high levels of phospholipids and the antioxidant astaxhantin. The stability of the EPA/DHA-normalized samples differed between the oils during the GI digestion, and the cod liver oil was the one most oxidized after completed digestion (highest [MDA] and [HHE]). In the comparison of the different oil digests, blanks with pure digests without added oil, were always included as controls.

When comparing the effects of the digested oils on HSP-70 and Trx-1 levels, a significant difference between the cod liver oil and the other two oils was observed; but no significant difference was observed between algae and krill oil. Digested cod liver oil did not significantly affect HSP-60, while both the digested algae and krill oils had a reducing effect; indicating that a low oxidation degree, as in the algae and krill oils after digestion, could have a HSP-60 decreasing effect. However, this effect could also be due to the protective effects from the natural antioxidants in the algae and krill oils [24,25,26].

Krill oil digests did not significantly reduce the levels of the superoxide dismutase SOD2 as the other oils did. Krill oil digests also had a lower HHE to MDA ratio than cod liver and algae oil digests, but if there is a specific effect of HHE on the down-regulation of SOD2 levels is not known. Free fatty acids (FFA) extracted from krill oil was previously found to inhibit cell growth and induce apoptosis when added in undigested form to HCT-15, SW-480, and Caco-2 cells [45]. Our digests contain a mixture of FFA, as well as partially hydrolyzed TAG and phospholipids, but indeed the FFA could play a specific role. In another study, undigested whole krill oil was also found to have anti-inflammatory action, thus reducing the pro-inflammatory cytokines IL-8 and TNFα that was produced by Caco-2 and HT29 cells [46]. In a human trial, krill oil was found to have a positive effect on intestinal endothelial function, giving a mean EndoPAT Reactive Hyperemia Index of 2.16 after 17 weeks of supplementation, and the ingestion of krill oil also increased the serum levels of high-density lipoprotein [47], however it is not known whether these results had any connection to formation of lipid oxidation products in the krill oil during digestion.

In general, our results from comparing the three different oil digests indicate that krill oil, cod liver oil, and algae oil, have similar characteristics in regards to not being cytotoxic or stress-promoting to intestinal epithelial cells. The oil digest with the most reducing effect on HSP-60, HSP-70, and Trx-1 was that from algae oil. Algae oil differed from the other oils in that it had a different FA profile with a high content of DHA, a low content of the other FA, and also another antioxidant profile. Whether these factors played a role in reducing the oxidative stress response requires future work.

## 5. Conclusions

Exposing the Caco-2 cells with digests of marine oils and pure aldehydes did not affect cell survival. All of the digests significantly reduced the cellular expression of the human cellular stress proteins HSP-70 and Trx-1, indicating that the cells were experiencing less oxidative stress. Exposure (*t* = 2 h) to pure MDA at the same level that was present in the digests (8.3 μM) significantly lowered the expression of SOD2. Corresponding exposure of pure HHE at the level found in the digests (1.4 μM) decreased expression of HSP-60 and Trx-1. A mix of MDA and HHE (45 μM of each) significantly diminished the cellular expression of Trx-1, however, at high levels (90 μM) there was no change in Trx-1 expression. Altogether, the present aldehyde concentrations, relevant to aldehyde levels formed in vivo, did not increase the levels of the investigated stress-related proteins, indicating that physiological levels of these aldehydes may not induce intestinal cell stress.

## Figures and Tables

**Table 1:** Proteins detected by the Human Cell Stress Array Kit.

Nr	Analyte	Nr	Analyte
**1**	ADAMTS1	**14**	IDO
**2**	Bcl-2	**15**	Phospho-JNK PAN (T183/Y185)
**3**	Carbonic Anhydrase IX	**16**	NFκB1
**4**	Cited-2	**17**	p21/CIP1
**5**	COX-2	**18**	p27
**6**	Cytochrome C (Cyt C)	**19**	Phospho-p38a (T180/Y182)
**7**	Dickkopf-4 (Dkk-4)	**20**	Phospho-p53 (S46)
**8**	Fatty acid-binding protein 1 (FABP-1)	**21**	Paraoxonase-1 (PON-1)
**9**	HIF-1a	**22**	Paraoxonase-2 (PON-2)
**10**	HIF-2a	**23**	Paraoxonase-3 (PON-3)
**11**	Phospho-HSP27 (S78/S82)	**24**	Thioredoxin-1 (Trx-1)
**12**	Heat Shock Protein-60 (HSP-60)	**25**	Deacetylase Sirtuin 2 (SIRT2)
**13**	Heat Shock Protein-70 (HSP-70)	**26**	Superoxide dismutase 2 (SOD2)

**Table 2:** Detected levels (μM) of 4-hydroxy-2-hexenal (HHE) and malondialdehyde (MDA) in in vitro digests (*t* = 210 min) using human digestive fluids. Data are shown as mean ± standard deviation (SD), *n* = 3.

Marine Oil	MDA μM ± SD	HHE μM ± SD
Algae oil	4.45 ± 1.81	0.13 ± 0.039
Cod liver oil	16.6 ± 7.74	2.77 ± 2.66
Krill oil	4.29 ± 0.70	0.38 ± 0.061

**Table 3:** Absolute quantity of 4-hydroxy-2-hexenal (HHE; nmol) present in the apical and basal medium (BM), *n* = 3.

HHE (nmol) Added	HHE (nmol), BM
0.7	0.0080 ± 0.0042
0.0325	0.0035 ± 0.0011

## Supplementary Materials

The following are available online at www.mdpi.com/2072-6643/9/11/1213/s1, Table S1: Amounts of EPA and DHA of algae oil, cod liver oil, and krill oil.
