# Supplementary material for: Effects of Marine Oils, Digested with Human Fluids, on Cellular Viability and Stress Protein Expression in Human Intestinal Caco-2 Cells
Source: Nutrients. 2017 Nov 4;9(11):1213. doi: 10.3390/nu9111213 (PMC5707685; doi:10.3390/nu9111213)
Supplement: Supplementary file 1 [file nutrients-09-01213-s001.pdf]

Supplementary Materials: Effects of Marine Oils, Digested with Human Fluids, on Cellular Viability and Stress Protein Expression in Human Intestinal Caco-2 Cells

Cecilia Tullberg, Gerd Vegarud, Ingrid Undeland and Nathalie Scheers

Table S1. Amounts of EPA and DHA (in % of total fatty acids) of algae oil, cod liver oil, and krill oil.

Oil	EPA (%)	DHA (%)	Total n-3 PUFA (%)	Phospholipids (%)	EPA (mg/g)	DHA (mg/g)	Total FAME (mg/g)
Algae oil	1	47	48	n.d.	7.7 ± 0.3	483.6 ± 15.6	847 ± 27.5
Cod liver oil	8.2	10.5	24.4	n.d.	76.9 ± 0.1	96.2 ± 0.5	735 ± 7.8
Krill oil	12.3	7.5	28.1	40	119.7 ± 1.2	60.6 ± 0.3	525.9 ± 10.8

% Data for algae oil and krill oil are according to the manufacturers specifications; cod liver oil data are according to Jónsdóttir *et al.* [33]. Quantitative data (mg FAME detected/g oil) according to Cavonius *et al.* [34]. Analysis with C17:0 as internal standard, and the fatty acid standard mix GLC 463 (Nu-Chek prep, Inc., Elysian, USA) as external standard. EPA, eicosapentaenoic acid; DHA, docosahexaenoic acid; PUFA, polyunsaturated fatty acids; FAME, fatty acid methyl esters.
